# Comparisons of drug-eluting balloon versus drug-eluting stent for the treatment of cancer patients presenting with acute myocardial infarction

**DOI:** 10.1186/s40001-023-01316-y

**Published:** 2023-09-09

**Authors:** Yi-Xing Yang, Huai-Peng Zhang, Chuang Li, Yuan Fu, Kui-Zheng He, Xin-Ming Liu, Hong-Jiang Wang, Kun Xia, Li Xu, Jiu-Chang Zhong, Mu-Lei Chen, Le-Feng Wang

**Affiliations:** 1 Heart Center and Beijing Key Laboratory of Hypertension, Beijing Chaoyang Hospital, Capital Medical University, No. 8, Gongti South Road, Chaoyang District, Beijing, 100020 China; 2 Department of Cardiology, Heze Municipal Hospital, No. 2888, Caozhou Road, Mudan District, Heze, 274000 Shandong China

**Keywords:** Cancer, Acute myocardial infarction, Percutaneous coronary intervention, Drug-eluting balloon

## Abstract

**Background:**

Treatment for cancer patients presenting with acute myocardial infarction (AMI) remains challenging. The objective of the study was to investigate the safety and efficiency of drug eluting balloon (DEB) versus drug eluting stent (DES) in this high-risk group.

**Methods:**

Between 1st January 2017 and 1st January 2022, cancer patients admitted to Beijing Chaoyang Hospital with AMI were retrospectively enrolled. The primary endpoint was major adverse cardiovascular event (MACE). The secondary endpoints included major bleeding events, heart failure and cardiac complications.

**Results:**

A total of 164 cancer patients presenting with AMI were included in the final analysis. Patients treated with DEB had a numerically lower rate of MACE than those treated with DES during a median follow-up of 21.8 months (22.9% vs. 37.1%, p = 0.23). Patients treated with DEB had a trend towards lower rate of major bleeding events than patients treated with DES (6.3% vs. 18.1%, HR 2.96, 95% CI [0.88, 9.92], p = 0.08). There were no significant differences between the two groups with regards to the rate of heart failure (4.2% vs. 9.5%, p = 0.32) and cardiac complications (0.0% vs. 2.6%, p = 0.56).

**Conclusions:**

The present study demonstrated that in cancer patients with AMI, DEB had a trend towards lower rate of major bleeding events and a numerically lower rate of MACE compared with DES.

**Graphical Abstract:**

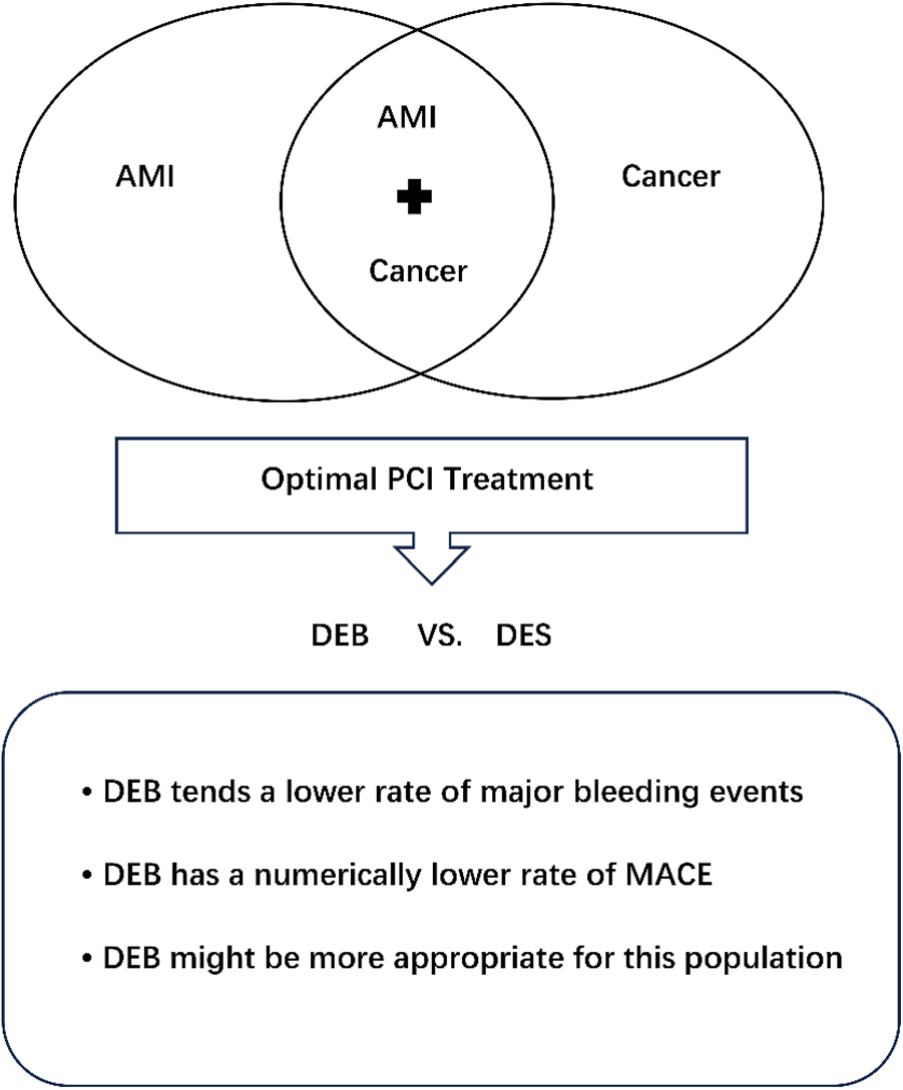

**Supplementary Information:**

The online version contains supplementary material available at 10.1186/s40001-023-01316-y.

## Introduction

Cancer patients with acute myocardial infarction (AMI) represent a particularly high-risk group as compared with general populations with AMI [1, 2]. Previous studies have demonstrated that AMI patients with current or historical diagnosis of cancer had significantly higher risks of all-cause mortality, major bleeding events and major adverse cardiac event (MACE) during both short- and long-term follow-up compared with those without cancer [1, 2]. Although percutaneous coronary intervention (PCI) with drug-eluting stent (DES) has become the preferred reperfusion strategy for patients with AMI [3], optimal management of cancer patients with AMI remains unclear because they are often excluded by large clinical trials due to the various comorbidities and complex scenario.

Indeed, despite significantly reducing the incidence of repeat revascularization, DES is associated with an increased risk of thrombotic complications due to the permanent vascular implants, and the prolonged dual antiplatelet therapy (DAPT) after implanting DES could lead to high risk of bleeding events [4, 5]. Therefore, the utilization of DES in cancer patients presenting with AMI who are paradoxically at high risk of bleeding as well as ischemic events might be limited. Considering the high morbidity and mortality in cancer patients presenting with AMI, identifying an effective therapy for this high-risk group is extremely required.

Drug-eluting balloon (DEB) is a novel treatment strategy that has emerged in recent years and has been proved to be safe and effective in treating patients with in-stent restenosis and has shown promising results in other indications such as small vessel disease, diffuse disease, bifurcations, chronic total occlusions and calcified complex lesions [6]. Theoretically, DEB could provide superiority over DES by avoiding the risk of stent thrombosis and allowing shorter duration of DAPT due to its ability of rapid local release of a high-concentration of antirestenotic drug into the coronary artery without using durable polymers and stent structures [6, 7], which makes DEB to be a potential therapeutic strategy in cancer patients presenting with AMI. The aim of the present study was to investigate the safety and efficacy of DEB compared with DES for cancer patients presenting with AMI.

## Methods

Between 1st January 2017 and 1st January 2022, patients admitted at Beijing Chaoyang Hospital with a diagnosis of cancer combined with AMI in were retrospectively enrolled. Patients were included if they met the following criteria: (1) age 18–85 years; (2) diagnoses of active cancer or historical cancer (active cancer was defined as receiving anticancer drug and/or radiotherapy, planning for undergoing cancer surgery, recurrent, metastatic, and/or inoperable cancer; cancer type included colon and rectum, stomach and esophagus, oral cavity and pharynx, hepatobiliary pancreas, prostate, kidney and bladder, lung, breast, uterus and ovary, blood, skin and leukemia and thyroid); (3) presenting with ST-segment elevation myocardial infarction (STEMI) undergoing primary PCI or presenting with non-ST-segment elevation myocardial infarction (NSTEMI) undergoing early invasive PCI (< 24 h from symptom onset); (4) receiving DES or DEB treatment during PCI. Patients were excluded if they met the following criteria: (1) cardiac arrest or cardiogenic shock; (2) mechanical complications; (3) not undergoing coronary angiography; (4) receiving medical treatment; (5) undergoing plain old balloon angioplasty (POBA), bare metal stent (BMS) or coronary artery bypass grafting (CABG); (6) severe coronary artery tortuosity, calcification and ectasia; (7) the combination use of DES and DEB in the target vessel segment. This study complied with the Declaration of Helsinki and has been approved by the Ethical Committee of Beijing Chaoyang Hospital.

The PCI procedure was performed according to current international guidelines and local practice. All patients were pretreated with aspirin 300 mg, clopidogrel 300 or 600 mg or ticagrelor 180 mg and intravenous heparin 70–100 IU/kg before procedure. Decisions regarding intervention strategy (DES or DEB), size of DES or DEB, inflation time and pressure of DES or DEB, administration of intra-aortic balloon pump (IABP), thrombus aspiration and glycoprotein IIb/IIIa inhibitor during the procedure were left to the discretion of the operator.

The primary endpoint of the present study was major adverse cardiovascular event (MACE), a composite of all-cause death, recurrent myocardial infarction (MI), acute stroke, or target lesion revascularization (TLR). The secondary endpoints included major bleeding events, heart failure and cardiac complications. Definitions of all-cause death, MI, stroke and TLR were corresponded with the Academic Research Consortium-2 Consensus document [8]. Major bleeding events were defined as Bleeding Academic Research Consortium [BARC] types 3–5 [9]. Heart failure was defined as any congestive heart failure [10]. Cardiac complications were defined as haemopericardium, cardiac tamponade, need for pericardiocentesis, and occurrence of coronary dissection. Clinical follow-up after the index procedure was conducted by reviewing hospital records, clinic visits and telephone interviews.

Continuous data were expressed as mean ± SD or median and interquartile range (IQR) and were compared using Student’s t test or Mann–Whitney rank-sum test. Categorical data were expressed as number and percentage and compared using Pearson’s Chi-square test or Fisher's exact test. Event-free survival was estimated with the Kaplan–Meier method and compared using the log-rank test and Breslow exact tests. Effect estimates for main outcome measures were calculated and are presented as hazard ratios and 95% confidence intervals using Cox proportional hazards models and compared using the Wald test. We also performed subgroups analysis for MACE to evaluate the consistency of treatment effects: age (≤ 65 vs. > 65 years); gender (male vs. female); cancer status (active cancer vs. historical cancer); AMI type (STEMI vs. NSTEMI); infarct-related artery (left anterior descending artery vs. non–left anterior descending artery); non-infarct related artery stenosis (≥ 70% vs. < 70%). A value of p < 0.05 was considered statistically significant. All statistical analyses were performed using SPSS 26.0 software (SPPS Inc., Chicago, IL, USA).

## Results

**Fig. 1:**
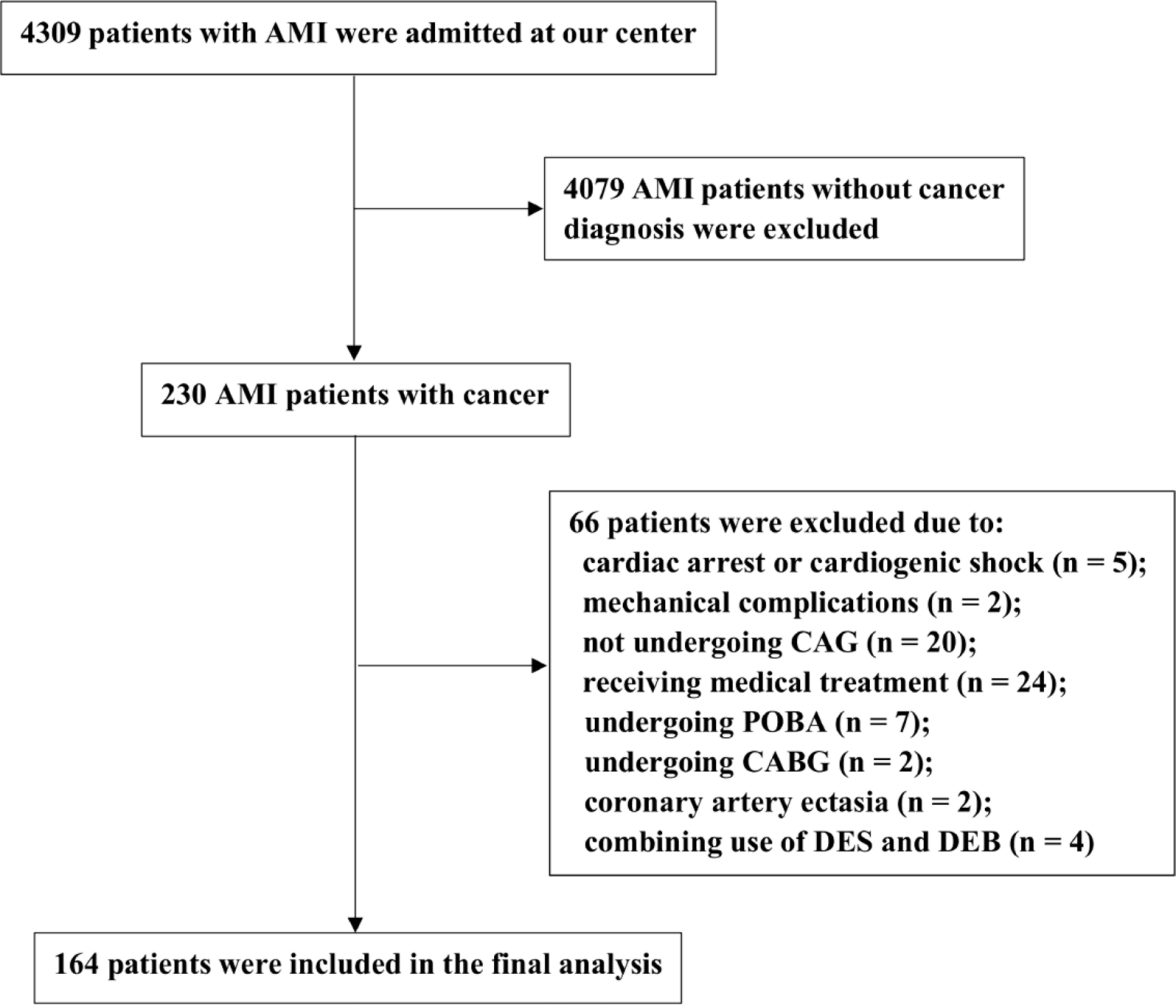
Patient flow chart. *AMI* acute myocardial infarction, *CAG* coronary angiography, *POBA* plain old balloon angioplasty, *CABG* coronary artery bypass grafting, *DES* drug-eluting stent, *DEB* drug-eluting balloon

Between January 2017 and January 2022, a total of 4309 patients with AMI were admitted at Beijing Chaoyang Hospital. Among them, 230 patients with AMI were identified with a diagnosis of cancer. Of these, 66 patients were not eligible due to the following reasons: cardiac arrest or cardiogenic shock (n = 5); mechanical complications (n = 2); not undergoing coronary angiography (n = 20); receiving medical treatment (n = 24); undergoing POBA or CABG (n = 9); coronary artery ectasia (n = 2); combining use of DES and DEB (n = 4). Thus, a total of 164 cancer patients presenting with AMI were included in the final analysis (Fig. 1). Among them, 48 patients were treated with DEB and 116 patients were treated with DES. Baseline demographic and clinical characteristics were comparable between the two treatment groups, except for a higher incidence of prior PCI and CABG (31.3% vs.18%, p = 0.02), a higher level of hemoglobin (137.40 ± 14.88 vs. 129.33 ± 22.04, p = 0.02) and a lower level of platelets at admission (189.75 ± 48.04 vs. 214.58 ± 62.13, p = 0.01) in the DEB group (Table 1). In addition, the DEB group had a lower frequency of LAD as infarcted related artery (33.3% vs. 50.9%, p = 0.04), less device number (1.0 [1.0, 2.0] vs. 2.0 [1.0, 2.0], p = 0.00), smaller device diameter (2.75 [2.50, 3.50] vs. 3.50 [3.00, 3.50], p = 0.00) and shorter device length (28.00 [20.00. 32.00] vs. 36.00 [24.00, 56.00], p = 0.00) than the DES group. DAPT duration was shorter in the DEB group than in the DES group (9.19 ± 5.31 vs. 11.28 ± 5.13, p = 0.02). No significant differences were observed with regard to other angiographic and procedural characteristics (Table 2).

**Table 1:** Baseline and clinical characteristics of patients

Characteristic	Overall (n = 164)	DEB group (n = 48)	DES group (n = 116)	p value
Age, years	68.05 ± 10.58	68.87 ± 10.48	67.72 ± 10.65	0.53
Male gender	114 (69.5)	35 (72.9)	79 (68.1)	0.54
BMI, kg/m^2^	24.80 ± 3.19	25.40 ± 3.65	24.56 ± 2.97	0.12
Hypertension	111 (67.7)	33 (68.8)	78 (67.2)	0.85
Diabetes mellitus	70 (42.7)	23 (47.9)	47 (40.5)	0.38
Hyperlipidemia	112 (68.3)	33 (68.8)	79 (68.1)	0.94
COPD	10 (6.1)	1 (2.1)	9 (7.8)	0.28
Chronic kidney disease	13 (8.0)	3 (6.3)	10 (8.7)	0.76
Stroke	22 (13.5)	9 (18.8)	13 (11.3)	0.21
Current smoking	103 (62.8)	33 (68.8)	70 (60.3)	0.31
Prior PCI and CABG	33 (20.1)	15 (31.3)	18 (15.5)	0.02
Family history of CAD	22 (13.4)	6 (12.5)	16 (13.8)	0.83
Cancer type				
Prostate, kidney, bladder	42 (25.6)	17 (35.4)	25 (21.6)	0.06
Gastrointestinal tract	38 (23.2)	10 (20.8)	28 (24.1)	0.65
Lung	31 (18.9)	11 (22.9)	20 (17.2)	0.40
Breast	16 (9.8)	5 (10.4)	11 (9.5)	1.00
Leukemia and lymphoma	12 (7.3)	3 (6.3)	9 (7.8)	1.00
Thyroid	9 (5.5)	1 (2.1)	8 (6.9)	0.29
Uterus and ovaries	8 (4.9)	1 (2.1)	7 (6.0)	0.44
Liver, cholecyst, pancreas	7 (4.3)	0 (0.0)	7 (6.0)	0.11
Others	1 (0.6)	0 (0.0)	1 (0.9)	1.00
Cancer status				
Active cancer	98 (59.8)	31 (64.6)	67 (57.8)	0.42
Receiving anticancer drug	40 (40.8)	10 (32.3)	30 (44.8)	0.24
Receiving radiotherapy	18 (18.4)	5 (16.1)	13 (19.4)	0.70
Historical cancer	66 (40.2)	17 (35.4)	49 (42.2)	0.42
Clinical presentation at admission				0.75
STEMI	72 (43.9)	22 (45.8)	50 (43.1)	
NSTEMI	92 (56.1)	26 (54.2)	66 (56.9)	
Admission haemodynamics				
Systolic blood pressure, mmHg	134.46 ± 22.93	139.83 ± 23.31	132.23 ± 22.49	0.05
Diastolic blood pressure, mmHg	76.11 ± 14.07	78.10 ± 16.04	75.28 ± 13.15	0.24
Heart rate, bpm	77.65 ± 14.64	77.02 ± 13.40	77.91 ± 15.17	0.73
Killip class at admission				0.43
1	134 (81.7)	41 (85.4)	93 (80.2)	
2–3	30 (18.3)	7 (14.6)	23 (19.8)	
LVEF, %	58.73 ± 10.27	58.92 ± 9.47	58.65 ± 10.63	0.88
Laboratory findings at admission				
TNI, ng/ml	3.50 (0.29, 28.51)	5.19 (0.05, 39.93)	3.32 (0.35, 38.51)	0.55
CKMB, ng/ml	7.65 (1.60, 38.10)	5.80 (1.60, 35.90)	7.80 (1.60, 42.35)	0.73
BNP, pg/ml	190.00 (76.00, 431.00)	148.00 (74.00, 446.00)	210.05 (84.00, 428.00)	0.41
ALB, g/l	39.98 ± 4.61	40.47 ± 4.99	39.78 ± 4.44	0.39
GLB, g/l	27.21 ± 4.97	27.31 ± 5.77	27.18 ± 4.62	0.88
TC, mmol/l	4.34 ± 1.12	4.20 ± 1.17	4.39 ± 1.09	0.32
TG, mmol/l	1.77 ± 1.78	2.10 ± 1.75	1.64 ± 1.16	0.13
HDL-C, mmol/l	1.00 ± 0.29	1.03 ± 0.34	0.99 ± 0.26	0.52
LDL-C, mmol/l	2.74 ± 0.97	2.58 ± 0.98	2.81 ± 0.96	0.15
UA, mmol/l	6.18 (5.08, 8.05)	6.44 (5.37, 7.97)	6.13 (4.96, 8.26)	0.39
CR, µmol/l	75.35 (63.60, 92.75)	74.90 (65.20, 93.40)	75.85 (61.60, 90.43)	0.57
GLU, mmol/l	8.07 ± 3.90	7.93 ± 3.83	8.13 ± 3.94	0.77
WBC, *10^9^/l	8.48 ± 4.08	8.21 ± 3.90	8.60 ± 4.17	0.58
HGB, g/l	131.69 ± 20.50	137.40 ± 14.88	129.33 ± 22.04	0.02
PLT, *10^9^/l	207.31 ± 59.30	189.75 ± 48.04	214.58 ± 62.13	0.01
d-dimer, mg/l	0.42 (0.27, 1.12)	0.48 (0.28, 1.51)	0.41 (0.27, 0.91)	0.48
FIB, mg/dl	340.41 ± 119.81	332.61 ± 89.79	343.64 ± 130.44	0.59

*BMI* body mass index, *COPD* chronic obstructive pulmonary disease, *PCI* percutaneous coronary intervention, *CABG* coronary artery bypass grafting, *CAD* coronary artery disease, *STEMI* ST-segment elevation myocardial infarction, *NSTEMI* non-ST-segment elevation myocardial infarction, *LVEF* left ventricular ejection fraction, *TNI* troponin I, *CK-MB* creatine kinase isoenzyme MB, *BNP* brain-type natriuretic peptide, *ALB* albumin, *GLB* globulin, *TC* total cholesterol, *TG* triglyceride, *HDL-C* high-density lipoprotein cholesterol, *LDL-C* low-density lipoprotein cholesterol, *UA* uric acid, *CR* creatinine, *WBC* white blood cells, *HGB* hemoglobin, *PLT* platelet, *FIB* fibrinogen

**Table 2:** Procedural and angiographic characteristics of patients

Characteristic	Overall	DEB group (n = 48)	DES group (n = 116)	P value
Infarct-related artery				
Left anterior descending coronary artery	75 (45.7)	16 (33.3)	59 (50.9)	0.04
Right coronary artery	50 (30.5)	18 (37.5)	32 (27.6)	0.21
Left circumflex coronary artery	33 (20.1)	14 (29.2)	19 (16.4)	0.06
Left main artery	6 (3.7)	0 (0.0)	6 (5.2)	0.18
In-stent restenosis	17 (10.4)	6 (12.5)	11 (9.5)	0.58
Bifurcation lesion	12 (7.3)	5 (10.4)	7 (6.0)	0.34
Small vessel disease	7 (4.3)	4 (8.3)	3 (2.6)	0.10
TIMI flow grade before PCI				0.71
0–1	136 (82.9)	39 (81.2)	97 (83.6)	
2–3	28 (17.1)	9 (18.8)	19 (16.4)	
Device characteristics				
Total number	1.0 (1.0, 2.0)	1.0 (1.0, 2.0)	2.0 (1.0, 2.0)	0.00
Total length, mm	33.00 (22.00, 50.00)	28.00 (20.00. 32.00)	36.00 (24.00, 56.00)	0.00
Maximal diameter, mm	3.50 (2.75, 3.50)	2.75 (2.50, 3.50)	3.50 (3.00, 3.50)	0.00
Maximal pressure, atm	13.32 ± 3.27	9.52 ± 1.38	14.90 ± 2.42	0.00
Thrombosis aspiration	18 (11.0)	5 (10.4)	13 (11.2)	0.88
Intra-aortic balloon pump	20 (12.2)	4 (8.3)	16 (13.8)	0.33
Glycoprotein IIb/IIIa therapy	35 (21.3)	10 (20.8)	25 (21.6)	0.92
TIMI flow grade post PCI				0.63
3	159 (97.0)	46 (95.8)	113 (97.4)	
0–2	5 (3.0)	2 (4.2)	3 (2.6)	
Non-infarct-related artery with stenosis > 70%	104 (63.4)	28 (58.3)	76 (65.5)	0.38
Intervention for non-infarct-related artery (n = 104)	45 (42.3)	13 (46.4)	32 (42.1)	0.69
Immediate complete revascularization (n = 45)	21 (47.7)	7 (53.8)	14 (43.8)	
Staged complete revascularization (n = 45)	24 (53.3)	6 (46.2)	18 (56.2)	
Doses of heparin, IU	6963.41 ± 1165.14	7000.00 ± 1275.80	6948.28 ± 1121.68	0.80
Medication at discharge				
Aspirin	162 (98.8)	47 (97.9)	115 (99.1)	0.50
Clopidogrel	152 (92.7)	45 (93.8)	107 (92.2)	1.00
Ticagrelor	13 (7.9)	4 (8.3)	9 (7.8)	1.00
Statins	158 (96.3)	46 (95.8)	112 (96.6)	1.00
Beta-blockers	108 (65.9)	31 (64.6)	77 (66.4)	0.83
ACEI/ARB	75 (45.7)	20 (41.7)	55 (47.4)	0.50
LMWH	117 (71.3)	31 (64.6)	86 (74.1)	0.22
PPI	66 (40.2)	16 (33.3)	50 (43.1)	0.25
DAPT duration, months	10.66 ± 5.26	9.19 ± 5.31	11.28 ± 5.13	0.02

*TIMI* thrombolysis in myocardial infarction, *PCI* percutaneous coronary intervention, *ACEI/ARB* angiotensin converting enzyme inhibitors/angiotensinogen type II receptor blockers, *LMWH* low-molecular-weight heparin, *PPI* proton pump inhibitor, *DAPT* dual antiplatelet therapy, *HR* hazard ratios, *CI* confidence intervals

**Fig. 2:**
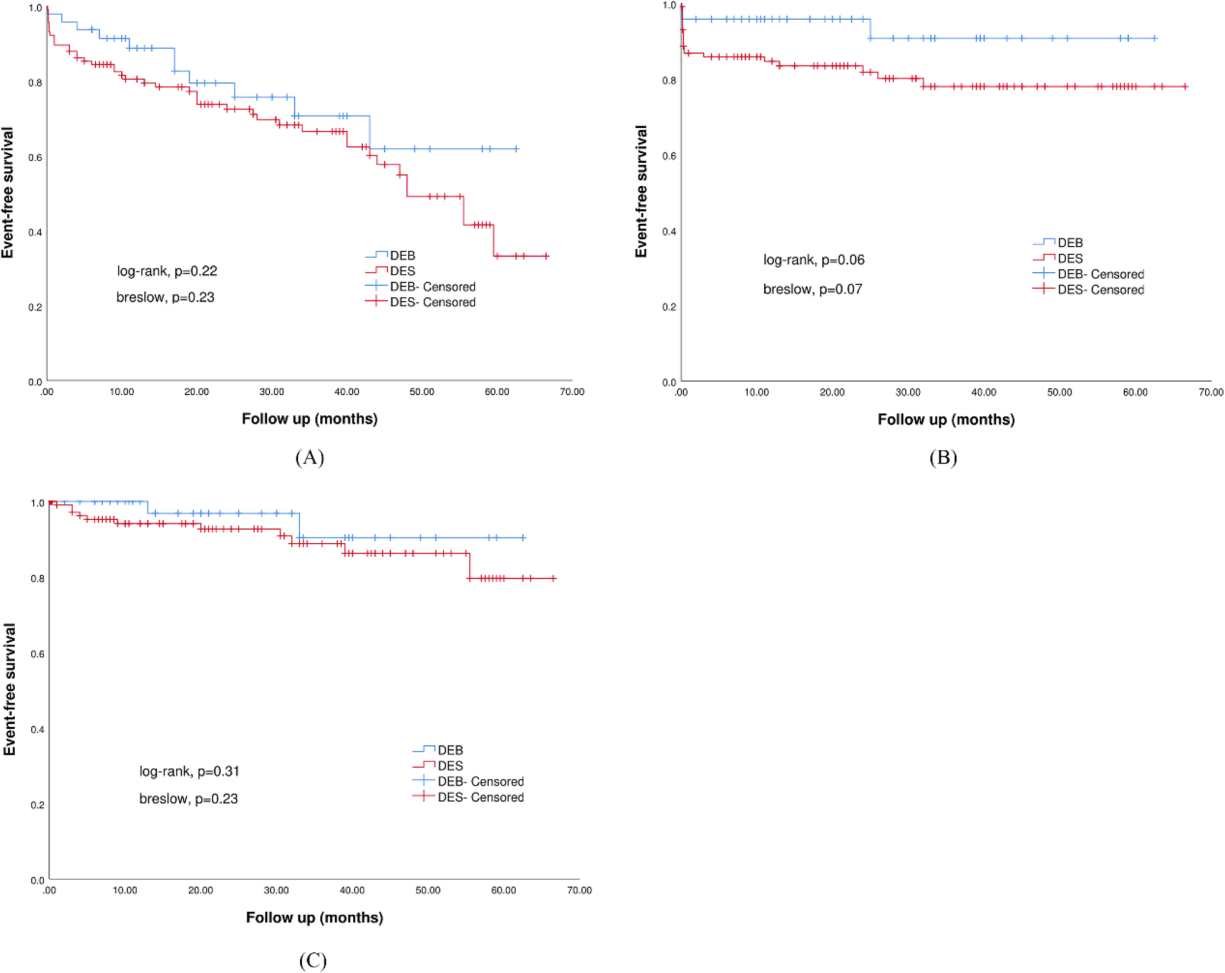
Kaplan–Meier survival curve for (**A**) major adverse cardiac events (MACE); (**B**) major bleeding events; (**C**) heart failure

Clinical follow-up data was obtained in all patients (Table 3). The median follow-up period was 20.0 (10.1, 33.4) months in the DEB group and 23.5 (8.6, 42.0) months in the DES group, respectively (p = 0.70). Patients treated with DEB had a numerically lower rate of MACE than those treated with DES during the follow-up period (22.9% vs. 37.1%, HR 1.50, 95% CI [0.77, 2.92], p = 0.23). No significant differences between the two groups were observed for the individual components of the composite outcome including all-cause death (16.7% vs. 20.7%, HR 1.14, 95% CI [0.51, 2.55], p = 0.75), MI (6.3% vs. 9.5%, HR 1.43, 95% CI [0.40, 5.15], P = 0.58), TLR (4.2% vs. 4.3%, HR 0.96, 95% CI [0.19, 4.94], p = 0.96) and stroke (2.1% vs. 6.9%, HR 3.15, 95% CI [0.39, 25.22], p = 0.28). Patients treated with DEB had a trend towards lower rate of major bleeding events than patients treated with DES (6.3% vs. 18.1%, HR 2.96, 95% CI [0.88, 9.92], p = 0.08). There were no significant differences between the two groups with regards to the risk of heart failure (4.2% vs. 9.5%, HR 2.16, 95% CI [0.48, 9.74], p = 0.32) and cardiac complications (0.0% vs 2.6%, p = 0.56). Kaplan–Meier survival curves for MACE, major bleeding events and heart failure were shown in Fig. 2A–C.

**Table 3:** Primary and secondary endpoints

	Overall (n = 164)	DEB group (n = 48)	DES group (n = 116)	HR (95% CI)	P value
Follow-up time, months	21.8 (9.0, 39.9)	20.0 (10.1, 33.4)	23.5 (8.6, 42.0)	N/A	0.70
Primary outcome					
MACE	54 (32.9)	11 (22.9)	43 (37.1)	1.50 (0.77, 2.92)	0.23
All cause death	33 (19.5)	8 (16.7)	24 (20.7)	1.14 (0.51, 2.55)	0.75
MI	14 (8.5)	3 (6.3)	11 (9.5)	1.43 (0.40, 5.15)	0.58
TLR	7 (4.3)	2 (4.2)	5 (4.3)	0.96 (0.19, 4.94)	0.96
Stroke	9 (5.5)	1 (2.1)	8 (6.9)	3.15 (0.39, 25.22)	0.28
Secondary outcomes					
Major bleeding events	25 (15.2)	3 (6.3)	21 (18.1)	2.96 (0.88, 9.92)	0.08
Heart failure	13 (7.9)	2 (4.2)	11 (9.5)	2.16 (0.48, 9.74)	0.32
Cardiac complications	3 (0.02)	0 (0.0)	3 (2.6)	N/A	0.56

*MACE* major adverse cardiac event, *MI* myocardial infarction, *TLR* target lesion revascularization, *HR* hazard ratios, *CI* confidence intervals

In the subgroup analyses, the results of comparison between the two groups were consistent across the six pre-specified subgroups (Additional file 1: Fig. S1), but DEB tended to be more effective than DES in terms of reducing MACE rate for patients with active cancer, despite no statistical significance was not achieved (16.1% vs. 40.3%, HR 2.57, 95% CI [0.97, 6.57], p = 0.07).

Multivariate regression analysis found that age (HR 1.04, 95% CI [1.01, 1.07], p = 0.02) and IABP (HR 4.51, 95% CI [1.99, 10.25], p = 0.00) were independently associated with MACE (Additional file 1: Table S1). In addition, DEB (HR 0.27, 95% CI [0.08, 0.94], p = 0.04) and age (HR 1.07, 95% CI [1.02, 1.12], p = 0.01) were independent predictors for major bleeding events (Additional file 1: Table S2). Furthermore, multifactor regression analysis showed no independent predictors for those encountered bleeding events after discharge (Additional file 1: Table S3).

## Discussion

To the best of our knowledge, this is the first study evaluating the safety and efficacy of DEB versus DES in cancer patients with AMI. The present study showed that as compared with patients treated with DES, those treated with DEB might benefit from a better clinical outcome, mainly driven by a trend towards lower rate of major bleeding events and a numerically lower rate of MACE. Moreover, the beneficial effects of DEB might be more apparent in patients with active cancer than in those with historical cancer.

In comparison to the general population, cancer patients are at an increased risk of both bleeding and ischemic events due to the haematologic, thrombocyte and coagulation abnormalities [11, 12], which may drive operators towards use a more conservative approach or to use POBA or BMS in this high-risk group. However, interestingly, an international study enrolling 15,401 patients with cancer and acute coronary syndrome (ACS) demonstrated that the use of POBA appeared to be related to an increased risk of adverse events at 1 year [13]. And another study including 6.5 million cancer patients with ACS showed that in-hospital mortality and major bleeding events were significantly greater in patients who received a BMS compared to those received a DES [14]. Despite of better outcomes achieved by DES, the rate of bleeding (6.4%) and complications (12.5%) during hospitalization remains higher [14]. Treatment for this high-risk group remains extremely challenging.

A DEB strategy might be superior over DES in some complex settings because it could provide a homogeneous distribution and high-concentration of antiproliferative drug and could rapidly release it into the coronary artery vessel without using durable polymers and stent structures, which could decrease chronic inflammatory response, delayed healing and hence a shorter DAPT [6, 7]. Previous large registry studies have shown excellent safety and efficacy of the DEB-only strategy for treatment of de-novo coronary artery lesions in patients with AMI. *Gobic *et al*.* concluded that in patients presenting with STEMI, there was a positive trend towards further reduction of MACE (0.0% vs. 5.4%, p = 0.29) and a significantly less late lumen loss (− 0.09 ± 0.09 vs. 0.10 ± 0.19, p < 0.05) at 6 months in the DEB treatment group than in the DES treatment group [15]. Additionally, the PEPCAD NSTEMI trial demonstrated that DEB compared to BMS or DES in treatment of patients with NSTEMI had a trend towards lower target lesion failure rate (3.8% vs. 6.6%, p = 0.53) and MACE rate (6.7% vs. 14.2%, p = 0.11) during a mean follow-up time of 9 months [16].

The DEBUT trial, which compared the safety and efficacy of DEB with BMS for de­novo lesions in patients at high bleeding risk, showed that the occurrence of MACE during 9 months follow-up was significantly lower in the DEB group than in the BMS group (1.0% vs. 14%, p < 0.00001) [17]. And there was no significant difference between the two groups with regards to the bleeding events (13.0% vs. 10.0%, p = 0.59). In a subgroup analysis of BASKET-SMALL 2 trial investigating the effect of DEB versus DES for the treatment of de-novo coronary small vessel diseases, DEB showed a similar safety and effectiveness as current-generation DES in the treatment of patients with high bleeding risk, with a similar MACE rate (18.6% vs. 15.4%) and major bleeding events (5.1% vs. 3.6%) at 3 years [18].

In accordance with the findings of these studies, the present study showed a favorable safety and efficacy of DEB in cancer patients with AMI, especially in those with active cancer. An analysis of a nationwide sample of US hospitalizations found that in STEMI patients undergoing PCI, 2.1% had a current cancer diagnosis and the rates of adverse events including MACE, all-cause mortality, and major bleeding were significantly higher in the active cancer groups compared to those without cancer [19]. A Japanese study showed that active cancer was present in 5.4% of patients with AMI undergoing PCI and patients with active cancer had an increased risk of MACE (35% vs.15.1%, p < 0.001) and major bleedings (10% vs. 3.8%, p < 0.001) compared with those with a history of cancer and no cancer during the mean follow-up period of 2.3 years [20]. Similarly, another Japanese study showed that 5.5% of patients with AMI undergoing PCI had an active cancer and the all-cause death (27.0% vs. 6.0%, p = 0.004) and major bleeding events (19.0% vs. 4.0%, p = 0.016) at 1 year were significantly higher in active cancer group than in the historical cancer group [21]. In line with previous studies, the present study found that 2.6% of patients with AMI undergoing PCI had an active cancer, However, there was no significant difference of MACE between the patients with active cancer and patients with historical cancer (32.7% vs. 33.3%, p = 1.00). One of the possible explanations was that in this study, patients who had completed curative surgical treatment or chemoradiation therapy within 1 year were categorized into the historical cancer group (a recent history of cancer), which might lead to a higher mortality rate in the historical group [22]. Interestingly, in the subgroup analysis, we found that the beneficial effects of DEB appeared to be more apparent in patients with active cancer than in those with historical cancer. Further large trials are required to investigate the effectiveness of DEB in active cancer patients.

The most common cancer diagnosis in the present study was urinary cancer (25.6%), followed by gastrointestinal tract (23.2%), lung (18.9%) and breast cancer (9.8%). We found that MACE rate during the follow-up period was the highest in the urinary group (35.7%), followed by lung (32.3%), breast (25.0%) and gastrointestinal tract (18.4%), and the bleeding rate was the highest in the urinary (19.0%) group, followed by lung (12.5%), breast (9.7%) and gastrointestinal tract (7.9%). However, a previous study demonstrated that AMI patients with gastrointestinal tract (28.6%) had the highest risk of cardiac death at 1 year after PCI treatment, followed by those with urinary (9.5%) and lung (9.1%) cancers, and the gastrointestinal tract (28.6%) had the highest risk of major bleeding, followed by urinary (14.3%) and lung (9.1%) cancers [21]. Another study investigating the in-hospital outcomes in patients who undergo PCI with a diagnosis of cancer according to cancer type found that the in-hospital mortality rate was the highest in ACS patients with lung cancer (4.2%), followed by colon (2.2%), breast (1.6%) and prostate (1.4%) after PCI treatment, and the bleeding rate was the highest in patients with a current diagnosis of colon (7.0%), followed by lung (6.9%), breast (5.1%) and prostate (3.8%) [14]. The discrepancy of clinical outcomes according cancer types between our study and previous studies could be partially explained by the lower incidence of MACE and bleeding rates in the DEB-treated gastrointestinal tract group (Additional file 1: Table S4). Besides, the exclusion of patients treated by POBA and BMS might also contribute to this different finding. Moreover, although there were no statistical differences, we found that patients treated with DEB had numerically lower rates of MACE and bleeding events than those treated with DES in each specific cancer group (Additional file 1: Table S4).

The choice and duration of dual antiplatelet therapy (DAPT) remains an intractable issue for cancer patients with AMI. A substudy of BleeMACS registry showed that in cancer patients presenting with ACS the single antiplatelet therapy with aspirin or clopidogrel was not related to the adverse events, while DAPT was demonstrated to be a protective factor (RR 0.5, 95% CI [0.3–0.9], p = 0.05) [13]. Besides, they found the new antiplatelet drugs (ticagrelor or prasugrel) seemed not to be significantly associated with the risk of bleeding and death. Therefore, DAPT with potent antiplatelet drugs might be safe and effective for cancer patients with AMI. Similarly, in the present study, multivariate regression analysis for those encountered bleeding events after discharge (excluding patients who had bleeding events during hospitalization) found that ticagrelor and DAPT duration were not independently associated with major bleeding events. Interestingly, when considering all patients encountered with bleeding events, DEB was shown as a protective factor for major bleeding events. Indeed, in line with previous studies [13, 20], the present study found that most bleeding events occurred during hospitalization, especially in patients treated with DES. One of the possible explanations was that systemic inflammatory response has been activated in earlier days (1–3 day) after DES implantation [23]. The cancer status might aggravate and accelerate this inflammatory response, which might further increase the bleeding risk.

According to the guidelines for AMI, the recommended duration of DAPT is 6 months in patients with ACS and stent implantation who are at high risk of bleeding, whereas in patients with AMI and high ischaemic risk who have tolerated DAPT without a bleeding complication, ticagrelor rather than clopidogrel or prasugrel for longer than 12 months on top of aspirin should be considered [3]. Besides, triple therapy with aspirin, clopidogrel, and an oral anticoagulant for longer than 1 month and up to 6 months should be considered in patients with high ischaemic risk due to ACS or other anatomical/procedural characteristics, which outweigh the bleeding risk. Dual therapy with clopidogrel 75 mg/day and an oral anticoagulant should be considered as an alternative to 1-month triple antithrombotic therapy in patients in whom the bleeding risk outweighs the ischaemic risk [3]. Therefore, antithrombotic treatment of patients with a cancer diagnosis should better be individualized on the basis of the bleeding and thrombotic risk. Hopefully, the DAPT score and PRECISE-DAPT score may serve as ideal tools to differentiate thrombotic versus bleeding risk in cancer patients, so important for decisions on the choice and duration of antithrombotic therapy [24]. Further studies are required to investigate the antithrombotic strategy in cancer patients with AMI.

## Limitations

The present study has several important limitations. First, it is a retrospective single-center study with an observational analysis, thus, the inherent biases cannot be completely avoided despite the adjustment for potential confounders using a multivariate regression model. Second, the results of should be interpreted with caution due to the small simple size in the present study. Besides, meaningful subgroup-analysis according to each cancer type could not be performed given the limited number of patients in each cancer subtype group. Third, this retrospective study lacked oral anticoagulation data. Further studies to investigate the antithrombotic therapy in cancer patients with AMI are required. Finally, the present study focused only on cancer patients with AMI who underwent PCI, data on those patients who receive conservative therapies were unavailable.

## Conclusions

The present study demonstrated that in cancer patients with AMI, DEB had a trend towards lower rate of major bleeding events and a numerically lower rate of MACE compared with DES. DEB might be safe and effective for treatment of cancer patients with AMI. Further large trials are required to investigate the effectiveness of DEB in cancer patients with AMI.

### Supplementary Information

**Additional file 1: Table S1**. Multivariate regression analysis of predictors for major adverse cardiac event. **Table S2**. Multivariate regression analysis of predictors for major bleeding events. **Table S3**. Multivariate regression analysis of predictors for major bleeding events after discharge. **Table S4**. Subgroup analyses stratified by the most common cancer types. **Figure S1**. Influence of the six pre-specified variables on the risk of MACE between the two treatment groups. All interaction tests were negative. HC: historical cancer; AC: active cancer; STEMI: ST-segment elevation myocardial infarction; NSTEMI: non-ST-segment elevation myocardial infarction; LAD: left anterior descending; NIRA: non-infarct related artery.

## Data Availability

The data that support the findings of this study are available from the corresponding author, Le-Feng Wang, upon reasonable request.

## References

[1] Moran TB, Plana JC (2020). Management of patients with acute coronary syndrome and cancer. Curr Cardiol Rep.

[2] Milazzo V, Cosentino N, Campodonico J (2020). Characteristics, management, and outcomes of acute coronary syndrome patients with cancer. J Clin Med.

[3] Neumann FJ, Sousa-Uva M, Ahlsson A (2019). 2018 ESC/EACTS Guidelines on myocardial revascularization. Eur Heart J.

[4] Kuramitsu S, Sonoda S, Ando K (2021). Drug-eluting stent thrombosis: current and future perspectives. Cardiovasc Interv Ther.

[5] Torrado J, Buckley L, Durán A (2018). Restenosis, stent thrombosis, and bleeding complications: navigating between scylla and charybdis. J Am Coll Cardiol.

[6] Jeger RV, Eccleshall S, Wan Ahmad WA (2020). Drug-coated balloons for coronary artery disease: third report of the International DCB Consensus Group. JACC Cardiovasc Interv.

[7] Buccheri D, Lombardo RM, Cortese B (2019). Drug-coated balloons for coronary artery disease: current concepts and controversies. Future Cardiol.

[8] Garcia-Garcia HM, McFadden EP, Farb A (2018). Standardized end point definitions for coronary intervention trials: the academic research consortium-2 consensus document. Eur Heart J.

[9] Mehran R, Rao SV, Bhatt DL (2011). Standardized bleeding definitions for cardiovascular clinical trials: a consensus report from the Bleeding Academic Research Consortium. Circulation.

[10] Ponikowski P, Voors AA, Anker SD (2016). 2016 ESC Guidelines for the diagnosis and treatment of acute and chronic heart failure: The Task Force for the diagnosis and treatment of acute and chronic heart failure of the European Society of Cardiology (ESC)Developed with the special contribution of the Heart Failure Association (HFA) of the ESC. Eur Heart J.

[11] Campia U, Moslehi JJ, Amiri-Kordestani L (2019). Cardio-oncology: vascular and metabolic perspectives: a scientific statement from the American Heart Association. Circulation.

[12] Al-Hawwas M, Tsitlakidou D, Gupta N (2018). Acute coronary syndrome management in cancer patients. Curr Oncol Rep.

[13] Iannaccone M, D'Ascenzo F, Vadalà P (2018). Prevalence and outcome of patients with cancer and acute coronary syndrome undergoing percutaneous coronary intervention: a BleeMACS substudy. Eur Heart J Acute Cardiovasc Care.

[14] Potts JE, Iliescu CA, Lopez Mattei JC (2019). Percutaneous coronary intervention in cancer patients: a report of the prevalence and outcomes in the United States. Eur Heart J.

[15] Gobić D, Tomulić V, Lulić D (2017). Drug-coated balloon versus drug-eluting stent in primary percutaneous coronary intervention: a feasibility study. Am J Med Sci.

[16] Scheller B, Ohlow MA, Ewen S (2020). Bare metal or drug-eluting stent versus drug-coated balloon in non-ST-elevation myocardial infarction: the randomised PEPCAD NSTEMI trial. EuroIntervention.

[17] Rissanen TT, Uskela S, Eränen J (2019). Drug-coated balloon for treatment of de-novo coronary artery lesions in patients with high bleeding risk (DEBUT): a single-blind, randomised, non-inferiority trial. Lancet.

[18] Scheller B, Rissanen TT, Farah A (2022). Drug-coated balloon for small coronary artery disease in patients with and without high-bleeding risk in the BASKET-SMALL 2 trial. Circ Cardiovasc Interv.

[19] Mohamed MO, Van Spall HGC, Kontopantelis E (2021). Effect of primary percutaneous coronary intervention on in-hospital outcomes among active cancer patients presenting with ST-elevation myocardial infarction: a propensity score matching analysis. Eur Heart J Acute Cardiovasc Care.

[20] Matsumoto T, Saito Y, Yamashita D (2021). Impact of active and historical cancer on short- and long-term outcomes in patients with acute myocardial infarction. Am J Cardiol..

[21] Tosaka K, Ishida M, Tsuji K (2021). Prevalence, clinical characteristics, and impact of active cancer in patients with acute myocardial infarction: data from an all-comer registry. J Cardiol.

[22] Hess CN, Roe MT, Clare RM (2015). Relationship between cancer and cardiovascular outcomes following percutaneous coronary intervention. J Am Heart Assoc.

[23] Li JJ, Yan HB, Xiang XP (2009). Comparison of changes in early inflammatory markers between sirolimus- and paclitaxel-eluting stent implantation. Cardiovasc Drugs Ther.

[24] Guo W, Fan X, Lewis BR (2021). Cancer patients have a higher risk of thrombotic and ischemic events after percutaneous coronary intervention. JACC Cardiovasc Interv.

