# Supplementary material for: Comparisons of drug-eluting balloon versus drug-eluting stent for the treatment of cancer patients presenting with acute myocardial infarction
Source: Eur J Med Res. 2023 Sep 9;28:334. doi: 10.1186/s40001-023-01316-y (PMC10492280; doi:10.1186/s40001-023-01316-y)
Supplement: Supplementary file 1 — Additional file 1: Table S1. Multivariate regression analysis of predictors for major adverse cardiac event. Table S2. Multivariate regression analysis of predictors for major bleeding events. Table S3. Multivariate regression analysis of predictors for major bleeding events after discharge. Table S4. Subgroup analyses stratified by the most common cancer types. Figure S1. Influence of the six pre-specified variables on the risk of MACE between the two treatment groups. All interaction tests were negative. HC: historical cancer; AC: active cancer; STEMI: ST-segment elevation myocardial infarction; NSTEMI: non-ST-segment elevation myocardial infarction; LAD: left anterior descending; NIRA: non-infarct related artery. [file 40001_2023_1316_MOESM1_ESM.docx]

**Table S1.** Multivariate regression analysis of predictors for major adverse cardiac event

| Variables | P value | HR | 95% CI |
| --- | --- | --- | --- |
| DEB | 0.47 | 0.73 | 0.31, 1.70 |
| Age | 0.02 | 1.04 | 1.01, 1.07 |
| Female | 0.89 | 0.96 | 0.49, 1.85 |
| Active cancer | 0.21 | 1.48 | 0.80, 2.73 |
| NSTEMI | 0.68 | 1.15 | 0.60, 2.23 |
| LAD | 0.35 | 0.73 | 0.38, 1.41 |
| Device number | 0.07 | 0.49 | 0.23, 1.05 |
| Device diameter | 0.88 | 1.05 | 0.56, 1.96 |
| Total length of device | 0.09 | 1.02 | 0.99, 1.05 |
| PLT | 0.74 | 1.00 | 0.99, 1.01 |
| HGB | 0.25 | 0.99 | 0.98, 1.01 |
| NIRA > 70% | 0.58 | 1.19 | 0.64, 2.20 |
| IABP | 0.00 | 4.51 | 1.99, 10.25 |
| Thrombosis aspiration | 0.56 | 0.73 | 0.25, 2.14 |

DEB: drug-eluting balloon; NSTEMI: non-ST-segment elevation myocardial infarction; LAD: left anterior descending coronary artery; PLT: platelet; HGB: hemoglobin; NIRA: non-infarct related artery; IABP: intra-aortic balloon pump; HR: hazard ratios; CI: confidence intervals.

**Table S2.** Multivariate regression analysis of predictors for major bleeding events

| Variables | P value | HR | 95% CI |
| --- | --- | --- | --- |
| DEB | 0.04 | 0.27 | 0.08, 0.94 |
| Age | 0.01 | 1.07 | 1.02, 1.12 |
| Female | 0.62 | 0.79 | 0.31, 2.02 |
| Active cancer | 0.76 | 0.87 | 0.35, 2.14 |
| NSTEMI | 0.60 | 1.27 | 0.53, 3.05 |
| LAD | 0.84 | 0.91 | 0.37, 2.24 |
| PLT | 0.68 | 1.00 | 0.99, 1.01 |
| FBG | 0.39 | 1.00 | 0.99, 1.01 |
| GPI | 0.15 | 1.99 | 0.78, 5.10 |
| PPI | 0.90 | 0.94 | 0.38, 2.33 |
| Ticagrelor | 0.53 | 1.66 | 0.35, 7.99 |
| Gastrointestinal tract | 0.06 | 0.23 | 0.05, 1.05 |

DEB: drug-eluting balloon; NSTEMI: non-ST-segment elevation myocardial infarction; LAD: left anterior descending coronary artery; PLT: platelet; FBG: fibrinogen; GPI: Glycoprotein IIb/IIIa therapy; PPI: proton pump inhibitor; HR: hazard ratios; CI: confidence intervals.

**Table S3.** Multivariate regression analysis of predictors for major bleeding events after discharge

| Variables | P value | HR | 95% CI |
| --- | --- | --- | --- |
| DEB | 0.47 | 0.44 | 0.05, 4.02 |
| Age | 0.20 | 1.07 | 0.97, 1.18 |
| Female | 0.69 | 0.70 | 1.21, 4.07 |
| Active cancer | 0.75 | 0.73 | 0.11, 4.96 |
| NSTEMI | 0.67 | 0.68 | 0.12, 4.01 |
| LAD | 0.41 | 2.24 | 0.34, 14.81 |
| PLT | 0.94 | 1.00 | 0.98, 1.02 |
| FBG | 0.56 | 1.00 | 0.99, 1.01 |
| GPI | 0.52 | 1.82 | 0.29, 11.31 |
| PPI | 0.91 | 0.90 | 0.13, 6.36 |
| Ticagrelor | 0.56 | 2.27 | 0.15, 34.77 |
| DAPT duration | 0.25 | 1.08 | 0.95, 1.22 |
| Gastrointestinal tract | 0.38 | 0.33 | 0.03, 3.88 |

DEB: drug-eluting balloon; NSTEMI: non-ST-segment elevation myocardial infarction; LAD: left anterior descending coronary artery; PLT: platelet; FBG: fibrinogen; GPI: Glycoprotein IIb/IIIa therapy; PPI: proton pump inhibitor; DAPT: dual antiplatelet therapy; HR: hazard ratios; CI: confidence intervals.

**Table S4. Subgroup analyses stratified by the most common cancer types**

**Major cardiac events**

| Cancer type | DEB | DES | p value |
| --- | --- | --- | --- |
| gastrointestinal tract | 1/10 (10.0) | 6/28 (21.4) | 0.65 |
| urinary | 5/17 (29.4) | 10/25 (40.0) | 0.48 |
| lung | 4/11 (36.4) | 6/20 (30.0) | 1.00 |
| breast | 0/5 (0.0) | 4/11 (36.4) | 0.25 |

**Major bleeding events**

| Cancer type | DEB | DES | p value |
| --- | --- | --- | --- |
| gastrointestinal tract | 0/10 (0.0) | 2/28 (7.1) | 1.00 |
| urinary | 3/17 (17.6) | 6/25 (24.0) | 0.72 |
| lung | 0/11 (0.0) | 4/20 (20.0) | 0.27 |
| breast | 0/5 (0.0) | 2/11 (18.2) | 1.00 |

**Heart failure**

| Cancer type | DEB | DES | P value |
| --- | --- | --- | --- |
| gastrointestinal tract | 1/10 (0.0) | 1/28 (3.6) | 1.00 |
| urinary | 1/17 (5.9) | 4/25 (16.0) | 0.63 |
| lung | 0/11 (0.0) | 2/20 (10.0) | 0.53 |
| breast | 1/5 (20.0) | 0/11 (0.0) | 0.31 |

**Figure S1.** Influence of the six pre-specified variables on the risk of MACE between the two treatment groups. All interaction tests were negative. HC: historical cancer; AC: active cancer; STEMI: ST-segment elevation myocardial infarction; NSTEMI: non-ST-segment elevation myocardial infarction; LAD: left anterior descending; NIRA: non-infarct related artery.
